# Management and Follow-Up Practices of Women With Recurrent Stress Urinary Incontinence Following Transobturator Mid-urethral Synthetic Sling Procedure: A 6-Year Retrospective Monocentric University-Based Study

**DOI:** 10.3389/fsurg.2021.710594

**Published:** 2021-09-03

**Authors:** Yi Huang, Zhengsen Chen, Baixin Shen, Yunpeng Shao, Jie Gao, Yiduo Zhou, Fisch Margit, Zhongqing Wei, Liucheng Ding

**Affiliations:** ^1^Department of Urology, Nanjing Medical University Second Affiliated Hospital, Nanjing, China; ^2^Department of Urology, Jiangnan University Affiliated Hospital, Wuxi, China; ^3^Department of Urology, Universitätsklinikum Hamburg-Eppendorf, Hamburg, Germany

**Keywords:** recurrent stress urinary incontinence, failed transobturator sling procedure, repeat retropubic sling procedure, urodynamic study, transperineal ultrasound

## Abstract

**Purpose:** The purpose of this study is to evaluate the efficacy of management and follow-up practices in repeat retropubic mid-urethral synthetic sling (MUS) procedure after transobturator tape/tension-free vaginal tape-obturator (TOT/TVT-O) failure, and to clarify the possible etiology of recurrent stress urinary incontinence.

**Methods:** The charts of all women patients who underwent tension-free vaginal tape (TVT) slings after previous failed transobturator MUS procedures between February 2012 and November 2018 at a single center were reviewed retrospectively. The transperineal ultrasound was performed to assess the pre-operative or post-operative urethral mobility and location of the slings. Furthermore, some essential evaluations were also made, mainly including medical history, physical examination, 1 h pad test, and urodynamic study. Finally, primary outcomes were evaluated according to the above items at 3, 6, and 12 months after the second operation, respectively.

**Results:** Thirty-five patients were included in the primary transobturator MUS sling procedure. At the 6 months follow-up, 32 (91.42%) patients were socially continent and negative in 1 h pad test. The transperineal ultrasound measurement results revealed that the bladder neck descent (BND) values were significantly decreased after the repeat sling operation, and better urinary continence function was observed according to the post-operative urodynamic study. Multifactorial etiologies resulted in recurrent stress urinary incontinence (SUI), including poor surgical technique, inadequate sling tension when treating ISD, and inappropriate sling position. Then the detail of the surgical procedure varied with the results of pre-operative evaluations, affecting the validity of the second sling.

**Conclusion:** Recurrent SUI has resulted from multi factors, pre-operative urodynamic study and transperineal ultrasound might be valuable tools to guide repeat sling operation and predict post-operative outcomes. A repeat TVT procedure may be regarded as a remedial measure for a failed transobturator MUS operation.

## Introduction

First described in the study conducted by Ulmsten et al., the mid-urethral synthetic sling (MUS) procedure has become the gold-standard surgical treatment for moderate to severe female stress urinary incontinence (SUI) with sustainable medium to long-term outcomes (1). However, in the long term, subjective cure rates range from 43 to 92% with a transobturator approach and from 51 to 88% with a retropubic approach, which is likely influenced by the surgical technique, the sling type, and adequate sling tension (2). It highlights the fact that practitioners are encountering increasing numbers of women with recurrent SUI after a failed MUS. In general, recurrent SUI following placement of an MUS imposes a challenge to surgeons, and it has been reported that the medium-term cure rates of repeat synthetic MUSs range from 60 to 70% which is lower than that achieved with primary surgery (3). At present, ideal evaluation and management of the women patients with recurrent SUI are therefore considered essential to the urological clinical practices, and a better understanding of SUI surgery failure.

From a theoretical standpoint, the implantation of MUS creates a firm insertion point for the pelvic floor muscles, restoring their contractile strength and closure, which is presumed to be an essential determinant of post-operative outcomes. While one may assume that a lack of sling tension may result in recurrent SUI, it is also hypothesized, on the other hand, that excessive pressure may favor urethral atrophy or sling erosion (4). Multiple investigative modalities should be applied in the clinic for the potential repeat MUS procedures, through which we promptly evaluate the pathological condition to formulate an individualized treatment. Meanwhile, a repeat MUS procedure in recent years is always considered a suitable remedial measure for a failed sling operation, with a high success rate of 71% during a 5-year follow-up (5). However, the evaluation before repeat sling operation is still uncertain nowadays. Furthermore, the transperineal ultrasound measurement for detecting the post-operative changes of pelvic floor anatomy in women with recurrent SUI is always overlooked, which may be very critical for our clinical decision-making (6).

In the clinic, apart from a thorough history and careful physical examination, the management of the women with recurrent SUI after MUS implantation generally includes functional assessment (such as urodynamic examination), cystourethroscopy, and Magnetic Resonance Imaging (MRI). Over the past decade, pelvic ultrasound has also become an essential diagnostic method in the evaluation of complications of MUS procedures which provides visual images on the dynamic changes of pelvic floor anatomy during functional tests (7). For these patients who may accept repeat MUS procedures, multiple investigative modalities may be necessary to fully evaluate the reasons for surgical failures and formulate an adequate treatment plan.

The objective of this study is to assess the application of urodynamic study and transperineal ultrasound imaging in women with recurrent SUI after transobturator MUS operations and introduce our management and follow-up practices for these complicated cases.

## Methods

### Study Design

The studies involving human participants were reviewed and approved by the Medical Ethics Committee of the Second Affiliated Hospital, School of Nanjing Medical University, reference number 2017-102. The patients/participants provided their written informed consent to participate in this study. After the approval of the Institutional Review Board (IRB), the charts of all women who underwent repeat retropubic MUS procedures between February 2012 and November 2018 at a single center were reviewed retrospectively for an exploratory study. Patients with a follow-up of <1 year were excluded. Moreover, neurological patients were excluded from the evaluation of pre-operative urodynamic study and nerve examination. All cases with primary MUS procedures through a transobturator approach [outside-in transobturator tape procedures [TOT] or inside-out tension-free vaginal tape-obturator [TVT-O]] were included. The mid-urethral synthetic sling (Herniamesh, Italy) was used in all cases.

### Pre-operative Evaluation

All patients underwent a complete evaluation before surgery, including the International Consultation on Incontinence Questionnaire-Urinary Incontinence Short Form (ICIQ-UI SF), 1 h pad test, urodynamic study, cystourethroscopy, and transperineal ultrasound. Meanwhile, the pre-operative assessment also comprised a detailed clinical interview and a physical examination. The physical examination was mainly focused on the assessment of vaginal surgical wound healing, vaginal prolapse, fistula, and urethral mobility.

Patients were then asked to conduct a 1 h pad test according to the International Continence Society (ICS) protocol (8). The grading is from 0 to 3, according to the standard: 0 (<1 g, negative), 1 (1–10 g, mild), 2 (10–50 g, moderate), and 3 (>50 g, severe). With grade 0 indicating that the patient is dry and grade 3 that the patient leaks all the time, irrespective of position or activity.

All urodynamic studies were performed with 7 F transurethral and rectal balloon catheters according to the ICS standards, which identified the type of SUI with the urodynamic parameters, including leak point pressure, bladder capacity, maximum detrusor pressure, maximum flow rate, and post-void residual volume.

After a complete emptying of the bladder, transperineal ultrasound imaging was performed on the women with MUS implantation.

### Principles of Transperineal Ultrasound Imaging of MUS

As performed entirely externally, the transperineal approach of ultrasound imaging is readily acceptable to most women patients and does not distort the pelvic organ anatomy compared with the transvaginal approach (7). The slings can be easily visualized on ultrasound in our clinical practices, and the position or tightness can be detected in the sagittal and axial view. Briefly, the transperineal ultrasound measurement was performed on these patients after bladder emptying (bladder volume is less than 50 ml) using a transperineal probe (3.5 MHz) in the sagittal and axial view, detecting the tape position or tightness. Meanwhile, the bladder neck and tape mobility at rest and Valsalva maneuver were detected through a 2D image measurement. Moreover, 3D transperineal ultrasound may be of particularly valuable for complex patients with multiple slings or other meshes.

**Figure 1:**
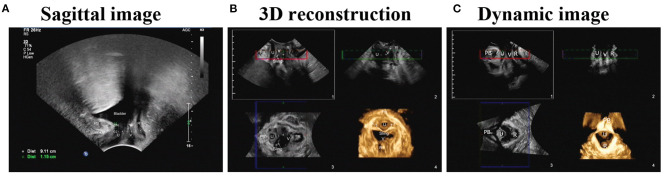
Real-time dynamic ultrasound images of the mobility of bladder neck, urethra and sling at rest or valsalva maneuver. **(A)** Sagittal transperineal ultrasound image demonstrating the anatomic structure of the pelvic. PB, Pubic bone, BN, bladder neck; U, urethra; V, vagina; R, rectum. **(B)** Axial 3D reconstruction and dynamic ultrasound technologies allows the assessment of sling and pelvic structures in multi imaging planes in a patient with MUS implantation through a transobturator approach. **(C)** Dynamic ultrasound images detected during valsalva maneuver or at rest in a female patient.

Ultrasound detection mainly provides real-time dynamic images of the mobility of bladder neck and sling, which can contribute to functional as well as anatomic assessment of sling problems. Briefly, making the pubic axis as a landmark to locate the lower edge of the pubic axis, urethra, anterior vaginal wall, and bladder neck, the distance between the sling midpoint and pubic symphysis was determined to assess the anatomic relationship of the sling and urethra. Afterward, patients were asked to do the Valsalva maneuver, simultaneously the movement of the urethra was detected, as well as the mobility of bladder neck and sling (Figure 1). Dynamic measurements were depicted three times for each patient.

### Repeat MUS Procedures

All repeat MUS procedures were performed by one surgeon (ZW) who had an experience of more than 1,000 cases of primary sling surgeries with the same type of polypropylene mesh. During the operation, we did not take out the previously placed sling with the absence of MUS-related complications. Then the detail of the surgical procedure varied with the results of the pre-operative evaluations.

For patients with mild to moderate SUI, an additional 2 cm polypropylene mesh sling was placed retropubically without tension at the mid-urethra. However, for patients with severe SUI or intrinsic sphincter deficiency (ISD), the implanted sling was adjusted with appropriate tension according to the real-time urine leakage condition of patients undergoing surgery (when they were asked to cough, sneeze, or do the maximal Valsalva).

### Post-operative Management

In suspected cases, cystourethroscopy should be performed to rule out bladder injury. The bladder catheter was removed at post-operative 48 h. Antibiotic therapy was applied for 48 h after operation. The patients attended outpatient clinics at 3, 6, and 12 months for follow-up. The follow-up visits involved a clinical interview, a physical examination, 1 h pad test, urodynamic study, and transperineal ultrasound.

### Outcomes of Interest

The primary endpoint was social continence at 6 months, defined as complete dry (1 h pad test, 1 g or less). Patients having a positive 1 h pad test (>1 g) were considered failures. Meanwhile, complications were identified from hospital and routine follow-up charts. A physician carried an examination of operative wounds and palpate for tape erosion at 12-month follow-up.

The other outcomes of interest were post-operative urodynamic study and transperineal ultrasound measurement. The post-operative urodynamic studies were carried out at 6 months, and the related parameters were collected to evaluate the urine control of the women patients. The post-operative transperineal ultrasound measurements were performed simultaneously, and the data were collected to assess the urethral mobility of these women.

The voiding diary data, post-operative lower urinary tract symptoms, and comorbidities were reported by the patients during the follow-up clinical interview.

### Statistical Analysis

Descriptive statistics (*M* and *SD*) were calculated for data. Changes in urodynamic parameters and ultrasound measurement data were compared between pre-operative and post-operative groups using *t*-tests.

Statistical analyses were performed using the SPSS 18.0 (SPSS Inc., Chicago, USA) statistical software. All tests were two-sided with a significance level at *P* < 0.05.

## Results

### Patient Characteristics

After the exclusion of seven patients with <-year follow-up, 35 patients met the inclusion criteria. The patient characteristics, operative details, and hospital stay are presented in Table 1. The mean age was 58.1 ± 13.2 years, and the median follow-up was 17 months. All 35 patients had a history of transobturator sling procedures at different medical institutions from Jiangsu province. None of the 35 patients had a history of pelvic surgery for recurrent SUI after failed transobturator MUS procedures.

**Table 1:** Patient characteristics, operative details, and hospital stay.

**Characteristic**	**Patients with failed TOT procedures**
Mean age (years)	58.1 (SD 13.2)
Mean BMI	22.9 (SD 2.6)
Post-menopausal	21 (60%)
Constipation	11(31.4%)
Nulliparous	0
Primary surgery time-point (month)	7.2 (SD 2.79)
History of other pelvic surgeries	0
*Material of previous slings*	
Synthetic sling	30 (85.71%)
Absorbable sling	3 (8.57%)
Unknown	2 (5.72%)
*Primary surgical approach*	
TVT-O	13
TOT	22
*Stress UI symptoms in past 7 days*	
No or small problems	3 (8.57%)
Yes, a big problem	31 (88.57%)
Unknown how much problem	1 (2.86%)
*Urge UI symptoms in past 7 days*	
No or small problems	27 (77.14%)
Yes, a big problem	7 (20%)
Unknown how much problem	1 (2.86%)
*Night time awakening to void in past 7 days*	
No or small problems	29 (82.86%)
Yes, a big problem	2 (5.71%)
Unknown how much problem	4 (11.43%)
*Preoperative 1 h pad test (g)*	44.2 (SD 11.57)
Negative (<1 g)	0
Mild (1–10 g)	0
Moderate (10–50 g)	29 (82.86%)
Severe (>50 g)	6 (17.14%)
*Urethrocystoscopy*	
Normal	35 (100%)
Pathological changes	0
*Operative details*	
Operative time (min)	24.7 (SD 5.6)
Blood loss (ml)	25.8 (SD 7.9)
TVT	35 (100%)
Hospital stay (d)	3.5 (SD 1.1)

### Patient Management and Follow-Up

**Figure 2:**
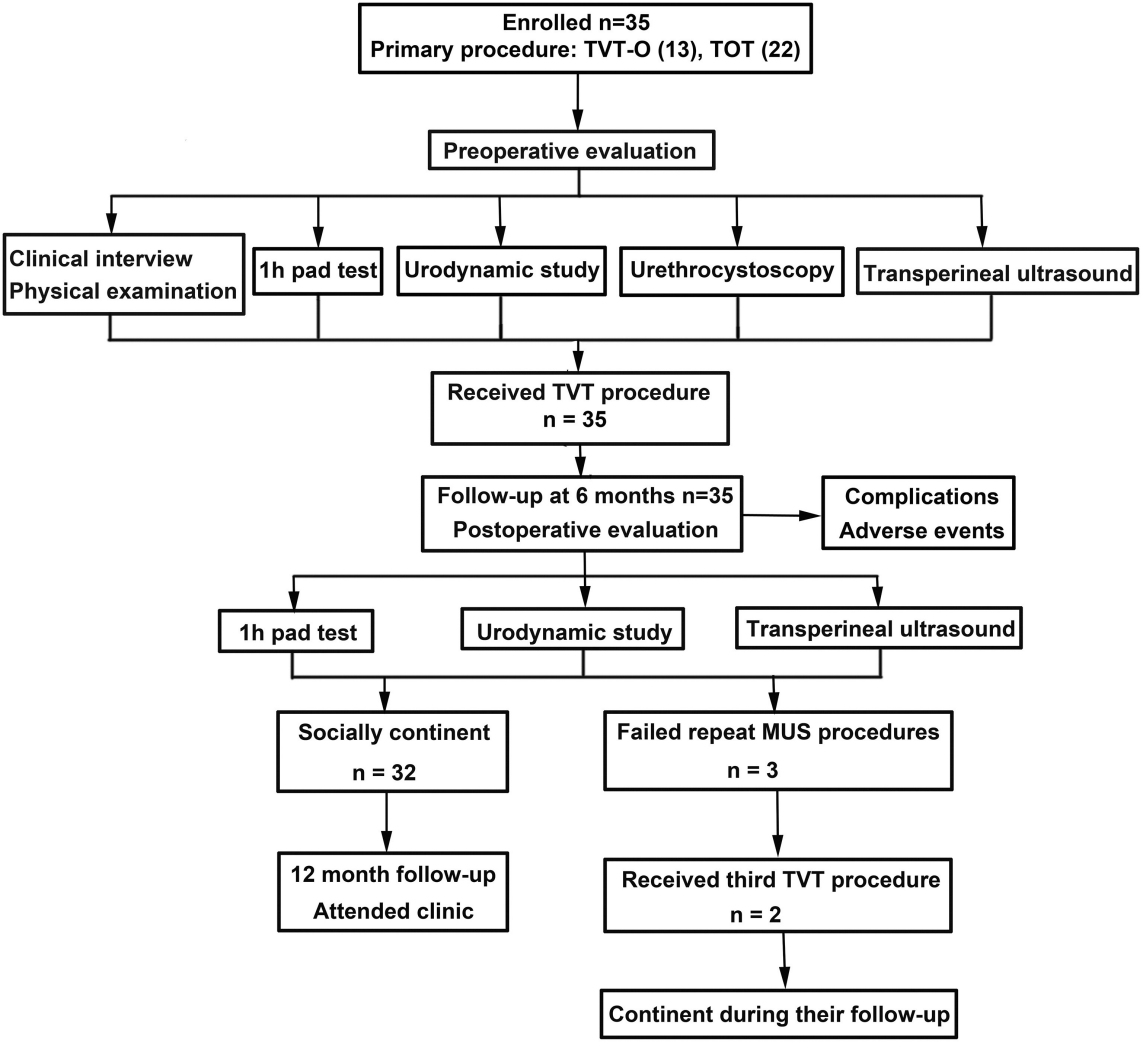
Flow of patient management and follow-up.

The pre-operative ICI-Q-SF score of these patients with recurrent SUI was 14.5 ± 3.6, and the post-operative score was 0.35 ± 1.4, suggesting that the post-operative urinary incontinence symptoms improved. Figure 2 shows a flow diagram of patient management and follow-up in the study. All the women patients received TVT procedures as their secondary operations. The overall post-operative complication rate during the follow-up visit was 7.5%, including 2 cases (8.6%) of urinary tract infection and 2 cases (5.7%) of urine retention. No women were admitted to the hospital during their follow-up.

The primary outcome, objective cure at 6 months post-operatively, was measured using a 1 h pad test in 35 women patients who received repeat MUS procedures. The results show that 32 out of 35 (91.42%) patients were socially continent and negative, 2 out of 35 (5.71%) patients had moderate SUI, and 1 out of 35 (2.86%) patients had severe SUI. Other pelvic procedures (a third TVT operation) were reported for two-thirds of the failed patients. Two patients reported an occasional burning sensation under the urethra. On vaginal examination, the majority of patients had normal palpation, but the sling was palpable (non-tender) for 6 (17.14%) of patients (Table 2).

**Table 2:** Six month follow-up.

	**Repeat MUS procedure**
*1 h pad test (primary outcome)*	
Negative (<1 g)	32 (91.42%)
Mild (1–10 g)	0
Moderate (10–50 g)	2 (5.71%)
Severe (>50 g)	1 (2.86%)
*Complications since hospital discharge*	
Additional pelvic procedures	
No	33 (94.29%)
Yes (third TVT)	2 (5.71%)
Surgery for mesh extrusion	0
Occasional urethral discomfort	2 (5.71%)
*Vaginal examination*	
Normal palpation	29 (82.86%)
Sling palpable but non-tender	6 (17.14%)

### Pre-operative and Post-operative Urodynamic Parameters

The urodynamic results of pre-operative maximum cystometric capacity (MCC) and post-operative MCC were 342.7 ± 53 and 364.6 ± 51.9 ml, respectively. The data of post-operative maximum flow rate (Qmax) (24.5 ± 5.8 ml/s) were significantly decreased (*P* = 0.0004) compared with pre-operative Qmax (30.1 ± 5.5ml/s). Meanwhile, there were significant differences (*P* = 0.0078) between pre-operative maximum urethral closure pressure (MUCP) (57.4 ± 19.6 cm H_2_O) and post-operative MUCP (74.3 ± 27.2 cm H_2_O). Subsequently, the data of post-operative abdominal leak point pressure (ALPP) (114.4 ± 24.1 cm H_2_O) were significantly increased (*P* = 0.0000) compared with pre-operative data (66.5 ± 22.9 cm H_2_O). All these data were shown in Table 3.

**Table 3:** Six-month follow-up urodynamic study results.

**Urodynamic parameters**	**Pre-operative data**	**Post-operative data**	***t*** **-test results**
MCC (ml)	342.7 (SD 53)	364.6 (SD 51.9)	*P* = 0.0966
Qmax (ml/s)	30.1 (SD 5.5)	24.5 (SD 5.8)	*P* = 0.0004
MUCP (cmH_2_O)	57.4 (SD 19.6)	74.3 (SD 27.2)	*P* = 0.0078
ALPP (cmH_2_O)	66.5 (SD 22.9)	114.4 (SD 24.1)	*P* = 0.0000

*MCC, maximum cystometric capacity; Qmax, maximum flow rate; MUCP, maximum urethral closure pressure; ALPP, abdominal leak point pressure*.

### Pre-operative and Post-operative Transperineal Ultrasound Measurement

Transperineal ultrasound provided a panoramic view of the pelvic organs without modifying the anatomical relationship between the urethra and the surrounding structural landmarks. Bladder neck descent (BND) is one of the reliable measurements used to assess urethral mobility in clinical practice (9). At rest, the pre-operative distance of bladder neck to symphysis pubis (BSD) was 2.61 ± 0.3 cm, post-operative BSD was 2.8 ± 0.37 cm. During Valsalva, pre-operative BSD was −0.35 ± 0.81 cm, post-operative BSD was 2.8 ± 0.37 cm. Then, we concluded that BND (3.04 ± 0.89 cm) was significantly decreased (*P* = 0.0000) after the repeat sling operation (1.09 ± 0.72 cm) (Table 4).

**Table 4:** Six-month follow-up transperineal ultrasound measurement results.

	**Pre-operative data**	**Post-operative data**	***t*** **-test results**
BSD (rest) (cm)	2.61 (SD 0.3)	2.8 (SD 0.37)	–
BSD (valsalva) (cm)	−0.35 (SD 0.81)	1.71 (SD 0.64)	–
BND (cm)	3.04 (SD 0.89)	1.09 (SD 0.72)	*P* = 0.0000

*BSD, distance of bladder neck to symphysis pubis; BND, bladder neck descent. BND = BSD (rest)-BSD (valsalva)*.

### Relationship Between Multiple Factors and Outcomes

**Figure 3:**
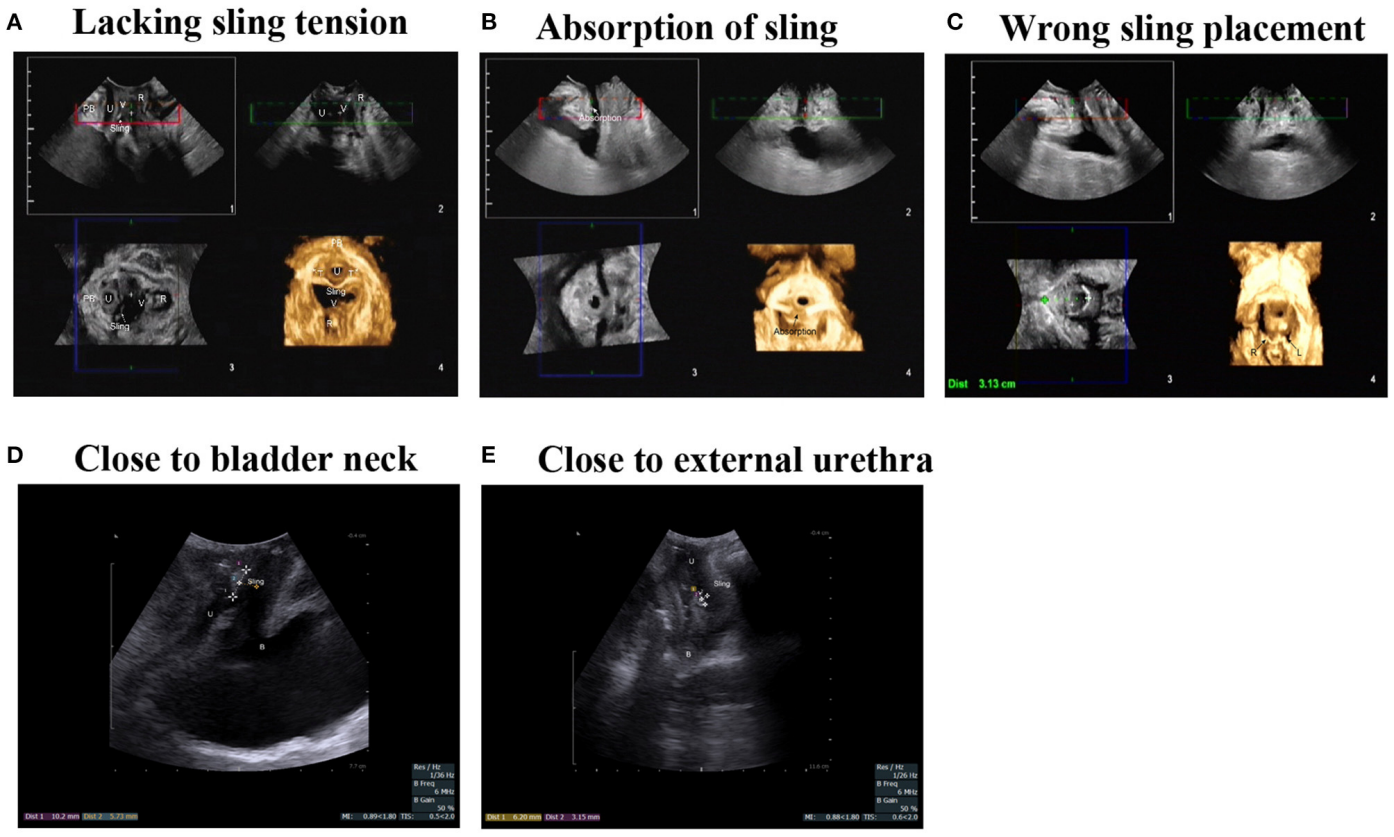
Failed transobturator MUS procedures with various reasons. **(A)** Insufficient urethral compression resulted from a lack of sling tension. **(B)** Absorption of absorbable sling leads to the weakening of urethral support. **(C)** The left TOT sling puncture path failed to pass through obturator. **(D)** Poor position of sling closing to bladder neck. **(E)** Poor position of sling closing to external urethra.

There are multiple factors concerning the post-operative outcomes of SUI patients with MUS procedures, mainly including sling tension and position. In this study, through visible transperineal ultrasound imaging, we could classify the exact reasons for the poor outcomes of these women patients, such as insufficient sling tension, absorption of the absorbable sling, and various poor sling position (Figure 3). All these factors resulted in the failure of the primary procedures to adjust the increased urethral mobility, which should be taken into consideration for repeat operations.

## Discussion

Stress urinary incontinence is a common condition affecting many women, caused by loss of support of the urethra. And sling procedure is considered effective in all types of SUI surgeries in the past decades. The cornerstone of post-operative success is to place the sling at the right position with an adequate tension that provides a hammock-like effect to prevent incontinence. However, ~31.5% of treated patients experience surgical failure with recurrent SUI, and 17% of them require repeat sling procedures. The main etiologies have been discussed in previous investigations, including poor surgical technique, inadequate sling tension when treating ISD (10), inappropriate sling position (11), and previous continence surgery. Furthermore, SUI patients with ISD using transobturator MUS may have more failure rate (12). Moreover, scarring and fibrosis of the primary operative area may gradually reduce the adequate sling tension in some cases. Furthermore, patients might have a coexisting sphincteric weakness that places them at greater risk of recurrence (12).

The primary aim of this study was to evaluate the pre-operative conditions of patients with failed MUS procedures and the results of repeated MUS procedures, representing our management and clinical practices for these complicated patients with recurrent SUI. So far, there is a paucity of literature discussing the clinical management of patients with failed MUS procedures (13). In the present study, we found a satisfactory success rate of repeat sling procedures using a retropubic suburethral sling procedure according to the detailed pre-operative evaluations, mainly including urodynamic study and transperineal ultrasound.

Transperineal ultrasound plays an important role in the evaluation of the patients with recurrent SUI following placement of a suburethral sling. Bladder neck descent is always considered to be positively correlated with the severity of SUI, and the data here confirmed that theory (Table 5). Moreover, the exact etiology can be identified using transperineal ultrasound, such as inadequate sling tension when treating ISD or inappropriate sling position (Figure 3).

**Table 5:** BND and the severity of SUI.

	**Moderate**	**Severe**	**Extremely severe**
BND	2.24 (SD 0.54)	2.16 (SD 0.65)	3.46 (SD 0.8)
1 h pad test (g)	4.63 (SD 2.16)	22.94 (SD 11.57)	158.09 (SD 54.66)

*BND, bladder neck descent*.

The effectiveness of the repeat sling appears to depend on adequate post-operative urethral mobility and urethral closure pressure which quantities of evidence suggest (14). Hence, adequate pre-operative evaluation on the urethral mobility, MUCP, and primary sling position might be essential for the clinicians to formulate the following treatment plan. Firstly, ultrasound imaging findings such as an open bladder neck or proximal urethra may suggest the presence of ISD (7). Nowadays, several studies reported that retropubic suburethral sling procedures had a better cure rate than transobturator procedures in patients with ISD (15, 16). Second, for patients with low MUCP (lower than 40 cm H_2_O) according to urodynamic studies, a repeat retropubic MUS procedure may be a suitable choice. Third, a hyper-mobile urethra detected using ultrasound imaging in patients with recurrent SUI means that the second sling procedure should be effective to adjust urethral mobility and midline position. At last, for patients with severe and extremely severe recurrent SUI, the sling should be placed at a certain position with sufficient tension to ensure enough MUCP and prevent sling movement.

For the position of the second sling, some recent studies have revealed that the success rate of repeated suburethral sling procedures was better for slings positioned at the proximal urethra (17). Meanwhile, urodynamic stress incontinence is more likely observed in women with a greater sling-pubis gap, and sling location should be close to the symphysis pubic to reduce the length of the urethral funneling (18, 19). Additionally, various parameters were always detected in the literature concerning SUI evaluation, including the distance of sling to the urethra, sling angle, and location of the sling relative to mid-urethral or bladder neck (20, 21). The results of the present study about these parameters also indicate that a narrower distance (~2.49 ± 0.21 cm) from the pubic symphysis to the sling midpoint is associated with de novo voiding dysfunction.

For the actual operation of the procedure, some detailed techniques may improve the final therapeutic effect. A remarkable improvement was observed in 31 out of 35 (88.5%) patients during a mean follow-up of 12 months. First of all, because of the scar formation and fibrosis in the previous operation area, we will not emphasize the separation of vaginal urethral space, just making enough gap for the placement of the second sling. Then, in the case of the short urethra (shorter than 3 cm), the sling should be stabilized at an appropriate mid location of the urethra to allow pressure transmission without movement.

The present study has limitations consistent with the retrospective design, although standardized documentation performed for the follow-up suggested a consistent quality of data, and the objective errors cannot be totally excluded. Moreover, the absence of patients with failed TVT procedures is another main shortcoming, and the patients with entire types of failed sling procedures cannot be totally investigated.

Finally, our management for these patients with failed transobturator slings may be referable in clinical practice, and the treatment for the failed sling patients will always be a challenge.

## Conclusion

The main reason for failed primary transobturator sling procedure in women patients often differ according to their certain post-operative conditions, including sling tension, sling position, and ISD. Repeat sling procedures may be reliable remedial measures for the women patients with recurrent SUI. According to our experience in the present study, the detail of the surgical procedure should vary with the results of pre-operative evaluations to ensure the validity of the second sling. Position and tension of the second sling were critical for post-operative outcomes, which were mainly determined in accordance with the transperineal ultrasound examination and urodynamic study. The present study might be regarded as a referable experience for future studies, which was needed to further elucidate the possible role of transperineal ultrasound measurements in the management of women patients with failed sling procedures.

## Data Availability Statement

The original contributions presented in the study are included in the article/supplementary material, further inquiries can be directed to the corresponding author/s.

## Ethics Statement

The studies involving human participants were reviewed and approved by the Medical Ethics Committee of the Second Affiliated Hospital, School of Nanjing Medical University, reference number 2017-102. The patients/participants provided their written informed consent to participate in this study.

## Author Contributions

LD: protocol development and manuscript writing. YH: manuscript writing and data analysis. ZC, JG, and YZ: data collection. BS and YS: data collection and data analysis. FM and ZW: protocol development and manuscript editing.

## Conflict of Interest

The authors declare that the research was conducted in the absence of any commercial or financial relationships that could be construed as a potential conflict of interest.

## Publisher's Note

All claims expressed in this article are solely those of the authors and do not necessarily represent those of their affiliated organizations, or those of the publisher, the editors and the reviewers. Any product that may be evaluated in this article, or claim that may be made by its manufacturer, is not guaranteed or endorsed by the publisher.
